# Essential of audiology: screening and post-screening

**DOI:** 10.1186/1824-7288-40-S1-A55

**Published:** 2014-08-11

**Authors:** Francesco Martines, Pietro Salvago, Salvatore Cocuzza, Eleonora La Mattina, Martinelli Stefano, Marianna Mucia, Enrico Martines

**Affiliations:** 1 Università degli Studi di Palermo, Dipartimento di Biomedicina Sperimentale e Neuroscienze Cliniche, (BioNeC), Sezione di Otorinolaringoiatria. Via del Vespro, 129 – 90127 Palermo, Italy; 2 Università degli Studi di Catania, Dipartimento di Specialità Medico-Chirurgiche, Clinica Otorinolaringoiatrica, Via Santa Sofia, 68, 95125 Catania, Italy; 3 Università degli Studi di Palermo, Dipartimento di Biopatologia e Biotecnologie Mediche e Forensi (Di.Bi.Me.F.), Sezione di Audiologia. Via del Vespro, 129 – 90127 Palermo, Italy; 4 Azienda Ospedaliera ‘Ospedale Niguarda Cà Granda’ – U.O.C. Neonatologia e Terapia Intensiva Neonatale. Piazza Ospedale Maggiore, 3 - 20162 Milano, Italy

## 

Newborn hearing screening is a type of screening test for the early detection of hearing loss. It can recognize with good accuracy newborns affected by hearing impairment allowing an early diagnosis and intervention and avoiding cognitive and linguistic deficits [1-6].

The incidence of bilateral sensorineural hearing loss (SNHL) in Sicily is 2.35 cases per 1000 newborns; this value increases to 2.95 if we consider also unilateral SNHL [2,3] and to 10 cases per 1000 births among infants at risk [7-9].

A correct newborn hearing screening programme is based on different protocols depending on the presence/absence of audiologic risk factors:

• Newborns without risk factors: [1-3]

**Initial Hearing Screening (Step I):** The initial screening should be performed using Transient-Evoked Otoacoustic Emissions (TEOAEs) in the birth centers as close to discharge as possible, preferably 12 hours or more after birth. It is recommended that an infant be referred for a re-screening (step 2) if s/he does not pass the initial screening or results cannot be obtained in one or both ears.

**Re-screening (Step II):** The re-screening should be performed in a second level center using TEOAEs and Automated Auditory Brainstem Response (AABR). If an infant does not pass the re-screening or if results cannot be obtained in one or both ears, s/he shall be referred to the regional third level center for diagnostic audiological evaluation.

• Newborns with risk factors (JCIH 2007) [7-10]

**Initial Hearing Screening (Step I)**: The Initial Hearing Screening should be performed in a second level center using TEOAEs and AABR. If an infant does not pass the initial screening or if results cannot be obtained in one or both ears, s/he shall be referred to the regional third level center for diagnostic audiological evaluation.

## Screening variables

Actually TEOAEs have a sensitivity of 100% and a specificity of about 70-95%. A higher TEOAEs specificity value depends on [1,2,11]:

• Timing of TEOAEs recording

• Trained and qualified personnel

• PASS/REFER criteria

## Limitations of screening

Audiologic screening does not identify:

• Post-natal SNHL (prelingual or perilingual) , mainly related to genetic causes [12,13]

• ANSD (Auditory Neuropathy Spectrum Disorder): the main risk factors associated to ANSD are severe jaundice, prematurity, respiratory distress, ototoxic drugs (used to treat neonatal infections), genetic mutations (e.g. OTOF gene). The diagnosis of ANSD is usually based on the combination of absent or abnormal ABR with normal TEOAESs and/or cochlear microphonics (CM) [14].
